# The Association of Vitamin D Status with Lipid Profile and Inflammation Biomarkers in Healthy Adolescents

**DOI:** 10.3390/nu12020590

**Published:** 2020-02-24

**Authors:** Amirhossein Yarparvar, Ibrahim Elmadfa, Abolghassem Djazayery, Zahra Abdollahi, Forouzan Salehi

**Affiliations:** 1 School of Nutrition, University of Vienna, 1090 Vienna, Austria; ibrahim.elmadfa@univie.ac.at; 2 Health and Nutrition Specialist for UNICEF Regional Office for Europe and Central Asia, Almaty 050000, Kazakhstan; 3 School of Nutrition and Dietetics, Tehran University of Medical Science, Tehran 14155/6117, Iran; jazaiers@tums.ac.ir; 4 Nutrition Department of the Ministry of health and Medical Education, Tehran 1467664961, Iran; abdollahi_z@yahoo.com; 5 Deputy Director of Family Health Department of the Ministry of health and Medical Education, Tehran 1467664961, Iran; salehi46@yahoo.com

**Keywords:** adolescent, Vitamin D status, Inflammation, lipid profile

## Abstract

Background: The association between vitamin D status and inflammatory biomarkers and lipid profile is not well known, especially in adolescents. Therefore, the aim of the current study is to investigate the association of vitamin D status with serum lipids and inflammatory biomarkers, including IL-10, IL-6, hsCRP, and TNFR-2, in male adolescents. Methods and materials: A sample of seventy-one high school male students, aged 17 years old, from a high school in Tehran were enrolled in the study. They were divided into four groups including group with serum vitamin D below 25 (ng/mL) (SVD < 25; *n* = 36), 25 and above (ng/mL) (SVD ≥ 25; *n* = 35), negative-hsCRP (*n* = 48), and positive-hsCRP (*n* = 23). Weight, height, body mass index, dietary intake, serum lipids, and inflammatory biomarkers, including IL-10, IL-6, hsCRP, and TNFR-2, were measured. Results: In the (SVD < 25) group, the serum level of TNFR-2 was significantly higher compared to that in the (SVD ≥ 25) group. There was a significant negative association between serum TNFR-2 and vitamin D levels in the whole sample. We found significant lower levels of IL-10 in positive-hsCRP group compared to the negative-hsCRP group. In addition, there was a significant negative correlation between the serum vitamin D level and hsCRP in both hsCRP groups. The HDL level was lower in the (SVD < 25) group compared to that in the (SVD ≥ 25) group. Finally, there was a negative correlation between the serum HDL and hsCRP levels in the positive-hsCRP subjects. Conclusion: Based on the findings it can be concluded that serum vitamin D affects HDL and inflammation status. Although serum levels of HDL and inflammation status are both predictors of metabolic syndrome and cardiovascular disease, further studies are needed to prove it, especially in adolescents.

## 1. Introduction

Vitamin D plays critical role in intestinal calcium absorption and is essential for maintaining skeletal integrity [1]. Recent studies also demonstrate that vitamin D has preventive roles in formation of chronic disease such as hypertension, cancer, and autoimmune diseases [2]. Studies over the last decade report that the vitamin triggers the immune cells to produce immunoglobulins; therefore, it enhances the strength of the immune system [3]. Vitamin D also decreases proinflammatory/anti-inflammatory ratio by switching the balance towards anti-inflammatory responses [4]. On the other hand, many studies showed that vitamin D contributes to joint and muscle comfort maintenance, and prostate, colon, and breast health [5]. It is well-known that severe vitamin D deficiency causes bone-related disorders such as rickets and osteomalacia [6]. On the other hand, secondary hyperparathyroidism due to vitamin D deficiency or insufficiency is an osteoporosis contributing factor [7,8]. Also, there has been a growing body of evidences for the role of vitamin D insufficiency in the initiation and progression of chronic diseases including cardiovascular diseases (CVD), hypertension, diabetes, autoimmune diseases, and cancers [2,9]. As most of these findings are from studies on adults, the vitamin D deficiency as an early onset risk factor in formation of chronic diseases during childhood and adolescence is less known.

Recent studies have shown that immunomodulatory effects of vitamin D are related to induction of interleukin 10 (IL-10). IL-10 is the most potent anti-inflammatory cytokine. Induction of IL-10 leads to inhibition of the both T-helper 1 (Th1) and Th2 activity [10,11]. IL-10 plays a pivotal role in controlling inflammatory processes, especially induction of oral tolerance, and its decrement results in numerous inflammatory responses, which are induced by enteric antigens [12]. The immunomodulatory effects of vitamin D are not limited to IL-10 induction; the negative association of IL-6 and high-sensitive C-reactive protein (hs-CRP) with vitamin D status have also been reported in previous studies [13,14]. Recent studies have demonstrated that inflammation, infectious, or noninfectious-related diseases can disrupt puberty processes and cause impairment in brain developmental [15,16]. The cytosolic domain of tumor necrosis factor-α receptor 2 (TNFR-2), after proteolytic degradation, releases into the circulation. In the circulation, TNFR-2 binds to the TNF-α, and can act as a reservoir for the cytokine [17].

Poor vitamin D status has been proposed to be associated with CVD. Vitamin D insufficiency is directly associated with mortality in CVD patients [18]. Several observational studies have indicated that improvement of vitamin D status decreases the risk of myocardial infarction and stroke [19,20,21]. It has been indicated that dyslipidemia is one of the important CVD risk factors. Previous works have proposed that there is an association between serum vitamin D levels and lipid profile [22,23]. Therefore, the vitamin D-related CVD risk reduction impact can be attributed to the improvement of vitamin D mediated dyslipidemia.

The results reported about the association of vitamin D status with inflammatory markers and lipid profile are inconsistent, especially in adolescents [24]. Therefore, the aim of the current study was to investigate the association of vitamin D status with serum lipids and inflammatory markers including IL-10, IL-6, hsCRP, and TNFR-2.

## 2. Material and Methods

In the present study, 71 third-grade high school male students, aged 17 years, from a high school in district 22 in Tehran, Iran were enrolled. The sampling was done between 28 September and 17 October of 2018. Exclusion criteria included cardiovascular disease (CVD), anemia, endocrine, hepatic, or renal disorders; malabsorption; and taking vitamin D supplement during the previous three months. The study was reviewed and approved by the Ethics Committee of Tehran University of Medical Sciences (No: IR.TUMS.VCR.REC.1396.4577), and written informed consents were obtained from all participants.

Weight and height of the students were measured with standard and calibrated scales to the nearest 0.1 kg and 0.5 cm, respectively. Body Mass Index (BMI) was calculated as weight (in kg) divided by height (in meters) squared. Assessment of dietary intake was performed using a valid and reliable food frequency questionnaire (FFQ) with 148 items [25]. The subjects were interviewed about their dietary intake during the previous day.

After an overnight fasting, blood samples were collected from the all participants, and the serum was separated by centrifuge and stored at −70 °C. ELISA kits were used to measure serum levels of IL-10 (Bioassay technology lab, Shanghai, China), IL-6 (Bioassay technology lab, Shanghai, China), TNFR-2 (Bioassay technology lab, Shanghai, China), hsCRP (Parsazmoon, Tehran, Iran), VLDL (Parsazmoon, Tehran, Iran), LDL (Parsazmoon, Tehran, Iran), TG (Parsazmoon, Tehran, Iran), and TC (Parsazmoon, Tehran, Iran). The absorbance was read using an Automatic ELISA Plate Reader. The 25 (OH) D was measured by an automated Roche electrochemiluminescence system; 25 ng /mL was considered as the cut-off for serum vitamin D [26], and serum levels of 1 mg/L level was considered as the cut-off for hsCRP [27]. This categorization has given enough sample size in each subgroup for sound statistical analysis

After measuring serum levels of vitamin D and hsCRP, the students were divided into the following four groups; group with serum vitamin D below 25 (ng/mL) (SVD < 25) (*n* = 36), group with serum vitamin D of 25 and above (ng/mL) (SVD ≥ 25) (*n* = 35), group with negative-hsCRP (*n* = 48), and group with positive-hsCRP (*n* = 23).

All the analyses were performed using SPSS 19.0 (IBM Corporation, Armonk, NY, USA). Based on the central limit theorem [28] and the results of Kolmogorov–Smirnov test, the data distribution was considered normal. Independent sample t-test and One-Way ANOVA were used to compare quantitative variables among the study groups. In addition, correlations between the measured variables were assessed using Pearson correlation. A *p*-values < 0.05 was considered to show statistical significance.

## 3. Results

The anthropometric, dietary, and biochemical variables of the study subjects are summarized in Table 1. The average BMI of the study subjects was 23.00 ± 1.89. As shown in the Table 1, 50.7% (*n* = 36) and 66.7% (*n* = 48) of the study subjects were vitamin D-deficient and hsCRP positive, respectively.

Table 2 shows the anthropometric, dietary, and biochemical variables of two vitamin D groups. The average serum vitamin D in both SVD < 25 and SVD ≥ 25 groups was 14.50 ± 3.70 and 31.98 ± 9.47, respectively (*p* < 0.001). There were no statistically significant differences among anthropometric, serum IL-10, IL-6, hs-CRP, VLDL, LDL, TC, and TG between the two vitamin D groups (*p* > 0.05). As shown in Table 2, the SVD < 25 group had significantly higher serum levels of TNFR-2 and lower serum level of HDL in comparison with SVD ≥ 25 group (*p* = 0.044 and *p* < 0.001, respectively). There was no significant difference between the groups in terms of dietary variables (*p* > 0.05).

As seen in Table 3, the average serum hsCRP in hsCRP positive and negative groups was 1.66 ± 0.653 and 0.500 ± 0.313, respectively (*p* < 0.001). There were no statistically significant differences in anthropometric indicators, serum IL-6, TNF-α, or lipid profile between the two groups (*p* > 0.05). The mean IL-10 in positive and negative hsCRP subjects was 108.20 ± 22.00 and 128.62 ± 31.27, respectively (*p* = 0.003). The differences between the two groups were statistically significant (Table 3). Also, dietary intake of energy and macronutrients were not statistically different (*p* > 0.05).

As shown in Table 4, height was negatively correlated with serum levels of vitamin D in (SVD < 25) subjects (*p* = 0.001; r = −0.615), whereas similar correlations were seen between the serum vitamin D and TNFR-2 in the total study population (*p* = 0.002; r = −0.367). A positive association was found between the serum levels of vitamin D and HDL (*p* < 0.001; r = 0.657). No significant correlation was found between serum vitamin D and the lipid profile components (except HDL) IL-10, IL-6, hs-CRP, weight, and BMI.

The results of correlation assessment between serum levels of hsCRP and anthropometric-, biochemical-, and lipid profile-related variables are summarized in Table 5. In negative hsCRP subjects, there was only a significant negative correlation between serum vitamin D and hsCRP (*p* = 0.020; r = −0.481); similar results were found in positive hsCRP subjects (*p* = 0.031; r = −0.311). The study results revealed that serum levels of hsCRP were indirectly associated with HDL (*p* = 0.002; r = −0.335) and marginally correlated with IL-10 (*p* = 0.070; r = −0.216). There were no significant associations between serum levels of hsCRP and other measured variables in the hsCRP groups.

**Figure 1:**
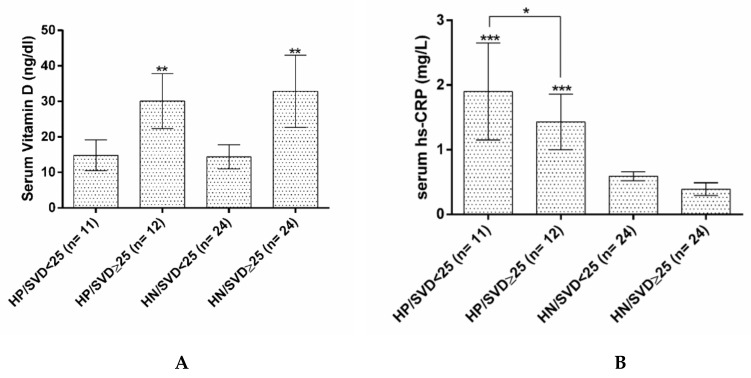
(**A**). Serum levels of hs-CRP in HP/SVD < 25, HP/SVD ≥ 25, HN/SVD < 25 and HN/SVD ≥ 25 groups. (**B**). Serum levels of vitamin D in HP/SVD < 25, HP/SVD ≥ 25, HN/SVD < 25 and HN/SVD ≥ 25 groups. HP/SVD < 25: hs-CRP positive/serum vitamin D< 25ng/mL; HP/SVD ≥ 25: hs-CRP positive/serum vitamin D ≥ 25ng/mL; HN/SVD <25: hs-CRP negative/serum vitamin D < 25 ng/mL; HN/SVD ≥ 25: hs-CRP negative/serum vitamin D ≥ 25ng/mL; hs-CRP: high sensitive C reactive protein. * *p*-value less than 0.05; ** *p*-value less than 0.05 vs. low vitamin D groups; *** *p*-value less than 0.05 vs. hs-CRP negative groups. Data analysis was done by ANOVA and Tukey post hoc tests.

On the other hand, the groups with hs-CRP positive and serum vitamin D of less than 25 ng/mL defined as HP/SVD < 25; *n* = 11, hs-CRP positive, and SVD ≥ 25 defined as HP/SVD ≥ 25; *n* = 12, hs-CRP negative and SVD < 25 defined as HN/SVD < 25; *n* = 24, and hs-CRP negative and SVD ≥ 25 defined as HN/SVD ≥ 25; *n* = 24 were assessed in terms of their lipid profile and inflammatory cytokines. As it is shown in Figure 1A,B, serum hs-CRP levels in HP/SVD < 25 was higher compared to HP/SVD ≥ 25 group (*p* < 0.05).

**Figure 2:**
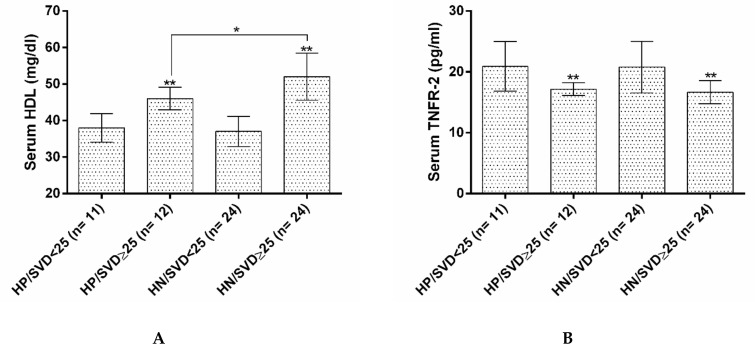

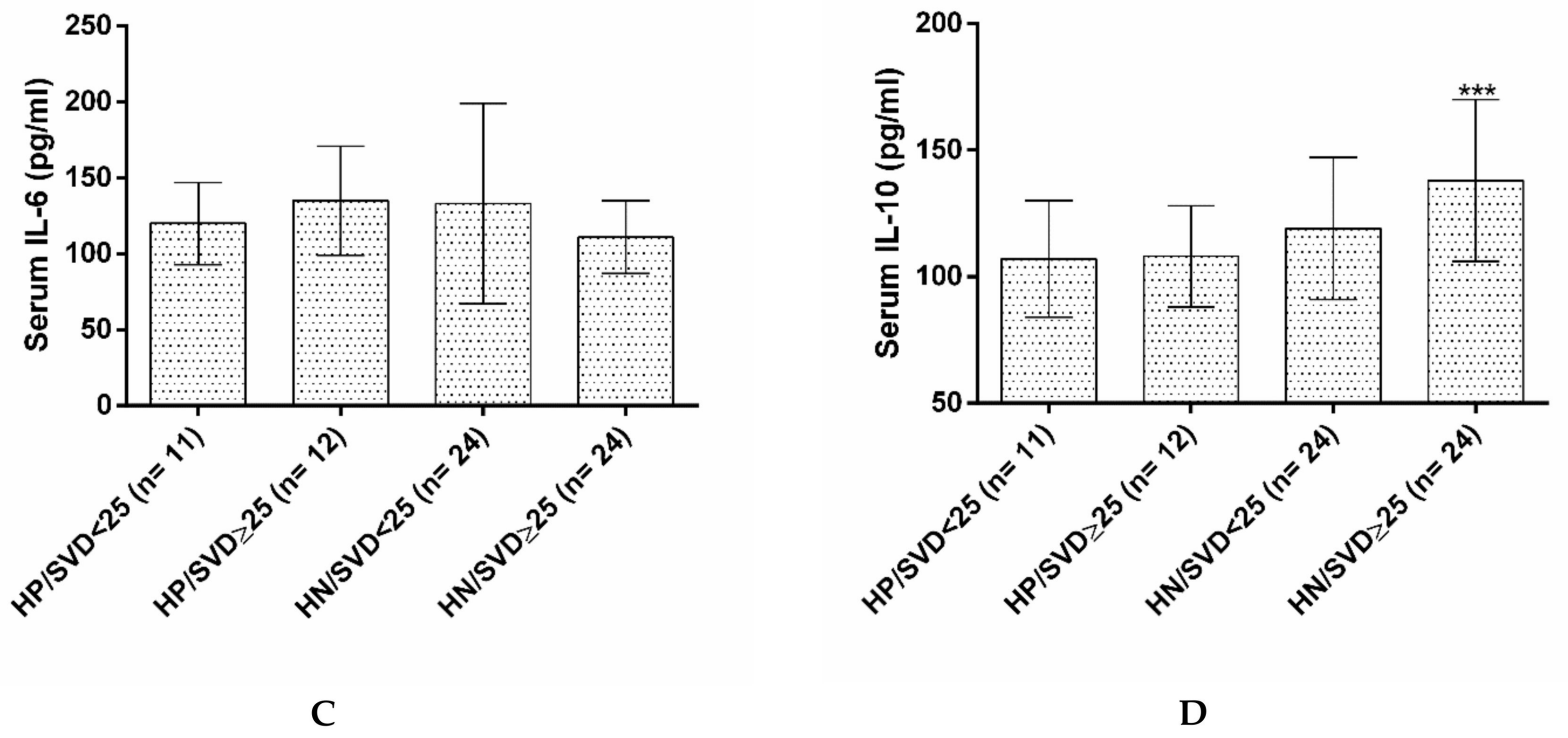
(**A**) Serum levels of HDL in four groups based on their Vitamin D and hs-CRP status. (**B**). Serum levels of TNFR-2 in four groups based on their Vitamin D and hs-CRP status. (**C**). Serum levels of IL-6 in four groups based on their Vitamin D and hs-CRP status. (**D**). Serum levels of IL-10 in four groups based on their Vitamin D and hs-CRP status. HP/SVD < 25: hs-CRP positive/serum vitamin D < 25ng/mL; HP/SVD ≥ 25: hs-CRP positive/serum vitamin D ≥ 25ng/mL; HN/SVD < 25: hs-CRP negative/serum vitamin D < 25 ng/mL; HN/SVD ≥ 25: hs-CRP negative/serum vitamin D ≥ 25ng/mL; IL: interleukin; HDL: high density lipoprotein; TNFR-2: tumor necrosis factor receptor 2. * *p*-value less than 0.05; ** *p*-value less than 0.05 vs. low vitamin D groups; *** *p*-value less than 0.05 vs. all other groups. Data analysis was done by ANOVA and Tukey post-hoc tests.

The serum TNFR-2 level was lower, but HDL was higher in SVD ≥ 25 groups compared to SVD < 25 groups. Also, within SVD ≥ 25 groups, subjects with negative hs-CRP had higher levels of HDL (*p* < 0.05). Serum IL-10 in the HN/SVD ≥ 25 group was significantly higher than the other groups (Figure 2A–D).

**Figure 3:**
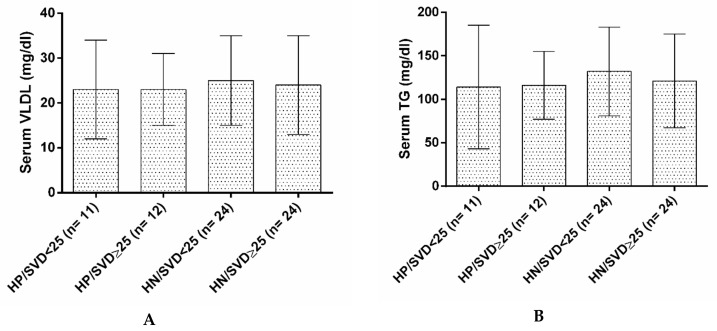

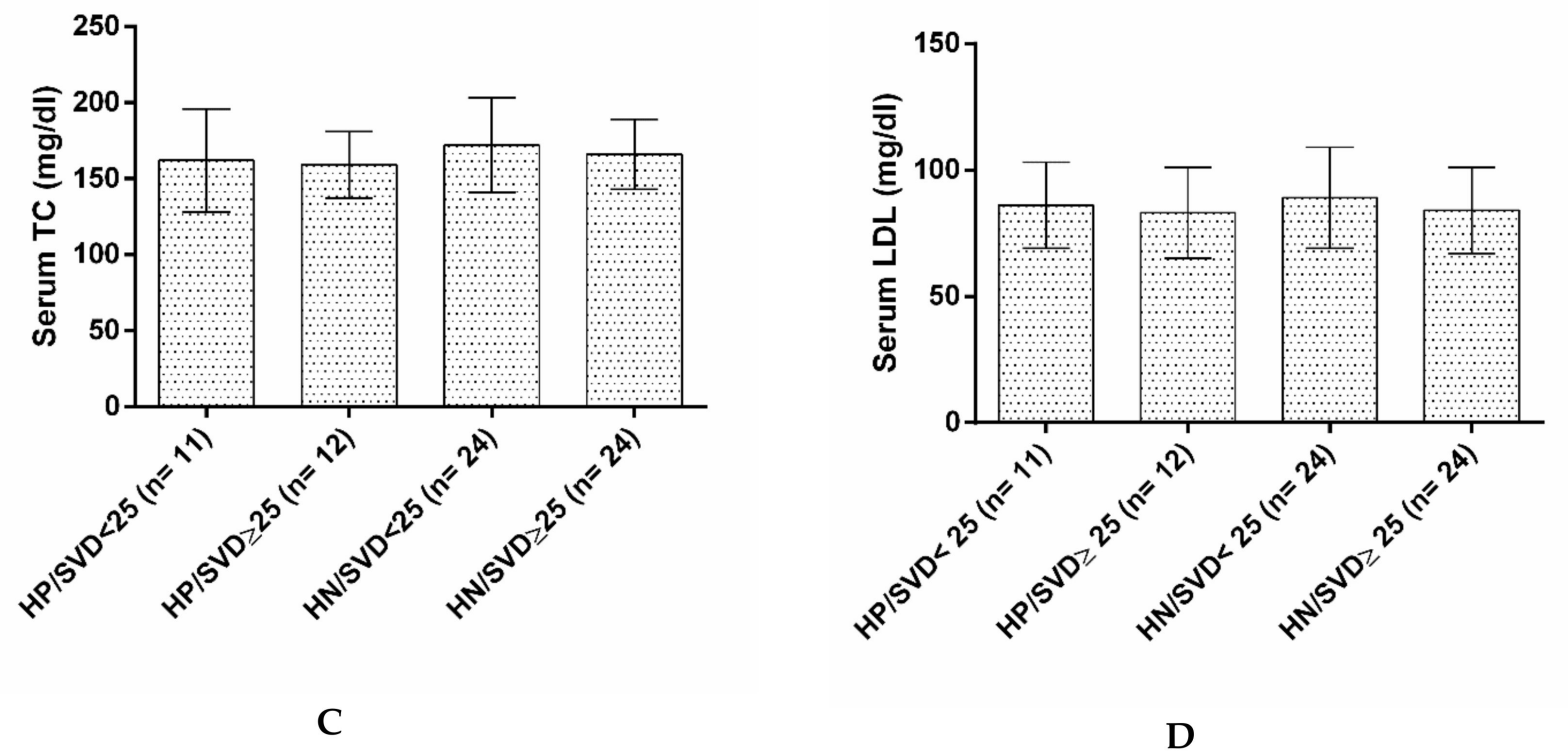
(**A**) Serum levels of VLDL in four groups based on their Vitamin D and hs-CRP status. (**B**). Serum levels of TG in four groups based on their Vitamin D and hs-CRP status. (**C**). Serum levels of TC in four groups based on their Vitamin D and hs-CRP status. (**D**). Serum levels of LDL in four groups based on their Vitamin D and hs-CRP status. HP/SVD < 25: hs-CRP positive/serum vitamin D < 25 ng/mL; HP/SVD ≥ 25: hs-CRP positive/serum vitamin D ≥ 25 ng/mL; HN/SVD<25: hs-CRP negative/serum vitamin D < 25 ng/mL; HN/SVD ≥ 25: hs-CRP negative/serum vitamin D ≥ 25 ng/mL; LDL: low density lipoprotein; TC: total cholesterol; VLDL: very low density lipoprotein. Data analysis was done by ANOVA and Tukey post hoc tests. No significant differences were obtained.

The results showed that serum levels of VLDL, LDL, TC, and TG were not affected by serum levels of hs-CRP or vitamin D (Figure 3A–D).

## 4. Discussion

In the present study, 71 high school male students aged 17 years were assessed for their vitamin D status (with the cut-off of 25 ng/mL of serum 25(OH)D as the threshold), and the association between their vitamin D status and serum lipids and inflammatory markers, including IL-10, IL-6, hsCRP, and TNFR-2, was examined. The findings showed that in male adolescents with SVD < 25, the serum level of TNFR-2 was significantly higher compared to SVD ≥ 25 group. Also, there was a significant negative association between serum TNFR-2 and vitamin D level in the study sample regardless of serum vitamin D or TNFR levels.

Studies on the association between TNFR-2 and vitamin D have often been in vivo or based on gene expression [29]. Tyler Barker et al. determined/assessed serum TNFR and vitamin D association in a randomized clinical trial (RCT) including vitamin D deficient, insufficient, and sufficient subjects in both the placebo and treatment groups. In that study, vitamin D supplementation (one bolus) in apparently healthy men was done with a dose of 100,000 IU [30]. At the end of the study, the results showed that in healthy male adults, supplementation of vitamin D could lead to decreases in the sTNFr2 level; however, there was no change in serum TNF-α. Also, the data suggested that soluble TNF receptors could be modulated in vitamin D sufficient, insufficient, or deficient subjects [30].

It has been suggested that TNF—a molecule with pleiotropic properties—has both stimulatory and inhibitory effects on bone resorption and bone formation, respectively [31,32,33]. TNFR2 has osteoclastogenic role and may facilitate or mediate role of TNF on osteoclastogenesis [34]. Although the importance of the role of TNFR2 in bone metabolism is not well defined, its ligands—including TNF-α and TNF-β—especially TNF-α—have an important role in inducing osteoclastogenesis [29].

On the whole, the results of the present study are consistent with those of previous studies on association between vitamin D and TNFR. It seems that vitamin D deficiency by increasing the TNFR-2 expression and activity could lead to an increased risk of inflammation.

On the other hand, we found significant lower levels of IL-10 in the positive hsCRP group in the present study as compared to the negative hsCRP group. Also, there were significant negative correlations between the serum vitamin D level and hsCRP in both positive and negative hsCRP groups.

Regarding the immune system, as it is known that the activated immune cells produce IL-10, which is a major anti-inflammatory cytokine [35], hsCRP indicates systemic inflammation and is a proinflammatory cytokine [36]. At a glance, it seems that higher levels of hsCRP in combination with lower levels of anti-inflammatory cytokines, such as IL-10, is an indication of inflammatory situation as Neslihan Seyrek et al. reported in the atherosclerotic hemodialysis patients [37].

Among cross-sectional studies in this regard, Doan T. et al., in their study on 253 normal people (51–77 years old), showed that low levels of 25(OH)D3 is negatively associated with the hsCRP level. Also, they found that plasma hsCRP had an inverse correlation with plasma 25(OH)D3 levels [38]. Ngo et al. showed that in healthy adults with a mean serum hsCRP level of 3.6 ± 4.0 mg/mL there was a significant inverse association between 25(OH)D and hsCRP concentrations [38]. Neng Chen et al. in their meta-analysis of randomized clinical trials about the effect of vitamin D supplementation on the circulating level of hsCRP, showed that vitamin D supplementation has beneficial effects, reducing circulating hsCRP levels. Also, they reported that this reduction was higher in populations with high circulating hsCRP levels [39]. However, H. Rahimi-Ardabili et al., in their RCT study on PCOS women, found that an oral dose of 150,000 IU vitamin D3/day for 2 months had no effect on serum hsCRP concentration [40].

The relationship between vitamin D status and inflammatory situation in different populations has been studied before, and in most cases the findings have shown protective anti-inflammatory effect of vitamin D. Our findings also support the previous results in this regard. Pivotal mechanism of the effect of vitamin D status on hsCRP levels is not understood yet but, based on the results from experimental studies, it seems that vitamin D3 can repress the release of TNF and suppress nuclear factor kappa-light-chain-enhancer of activated B cells (NFKB), both of which are important and active components of inflammation [38].

Altogether it seems that lower vitamin D levels (such as in persons with an insufficient or deficient status) may lead to higher hs-CRP and lower anti-inflammatory cytokine IL-10. This inflammatory situation could activate TNFR and cause more bone loss or even lead to shorter height in adolescent males, as we found a negative correlation between the serum levels of vitamin D in the vitamin D-deficient subjects in the present study.

The findings of the present study showed lower levels of HDL in the low vitamin D level group as compared to the high vitamin D level group. Also, we found a negative correlation between the serum HDL and hsCRP levels in positive hsCRP subjects. H. Rahimi-Ardabili et al. in their RCT study on polycystic ovary syndrome (PCOS) patients reported that supplementation of 150,000 IU vitamin D per day for two months could lead to a significant increase in the serum vitamin D level and significant decreases in lipid profile components including serum TC, TG, and VLDL; however, it had no significant effects on the serum HDL and LDL levels [40]. Kelishadi R et al., in their meta-analysis that included seventeen studies with more than 25,000 participants, investigated the association of vitamin D status and lipid profile in children and adolescents. They concluded that all of the studies (15 studies) except one [41] showed a direct positive association between the serum 25-(OH)D and HDL levels [24].

Many mechanisms were reported for the influence of vitamin D on lipid profile, but how vitamin D affects the profile is unknown yet. Increasing intestinal absorption of calcium could decline production and release of liver-originated TG [42]. Vitamin D could reduce production and release of TG through induction of intestinal absorption of calcium. Increment in absorption of calcium through gastrointestinal tract could decline absorption of fats due to the insoluble nature of calcium-fatty complexes in which formed during digestion [43]. Calcium promote the synthesis of bile acids from cholesterol and thereby decline serum cholesterol [44]. Vitamin D suppress serum parathyroid hormone (PTH) [45,46]. High PTH levels could result in increment of TG production. Β cell function impairment caused by vitamin D deficiency is another mechanism for possible mechanism for our results. Insulin resistance leads to TG increment and HDL decrement [47,48,49]. Vitamin D directly, and not through calcium, induces synthesis of bile salts [50]. On the other hand, it has been suggested that proinflammatory cytokines such as (TNF)-a, IL-6, and IL-1, which are the major cytokines in the inflammatory responses, could affect synthesis of apolipoproteins [51]. These responses originate from proinflammatory cytokines leading to lipolysis in fatty tissues and production of hepatic fatty acids, followed by increases in TG and VLDL levels [52]. Although the exact mechanism of HDL reduction in inflammation is not clear [53], it has been suggested that reduced activity of lecithin-cholesterol-acyl-transferase (LCAT), which occurs in inflammation, may decrease cholesterol esters formation, which will affect the formation of mature HDL particles [54].

Regarding the threshold to categorize vitamin D status, globally, a wide “optimal” range for serum 25(OH)D is reported (25–80 ng/mL), where some variation is related to geographical and epidemiological differences [55]. Although the major diagnostics manufacturers have recently developed improved automated tests for Vitamin D, the intra- and inter-laboratory variability is still high which might lead to incorrect vitamin D deficiency/insufficiency diagnosis. Thus, studies have suggested that a desirable range for vitamin D should be calculated using a validated equation that takes into account the UVB-component, ethnicity, BMI, age, sex, and eventually vitamin D supplementation [56]. Although devising and using such equations was not in the scope of our study, based on a qualitative assessment of the experts’ views, considering the geographical, epidemiological, age group, and ethnical factors, it was recommended to use the 25 ng/mL as the cut-off in our study. In addition, the proposed threshold of 20, 21–29, and 30 ng/mL 25 (OH) D is mainly derived from studies on role of vitamin D on calcium homeostasis, bone mineralization, and PTH levels, whereas the consequences of vitamin D deficiency on organs other than bone are not yet fully known [55]. Having said this, there are still a number of studies that have identified 25 ng/mL as a better cut-off to differentiate the bone mineral density [57]. Using the cut-off of 25 ng /mL also enabled us to obtain a better and adequate distribution of samples in all four subgroups of the study. The sample size for all subgroups would have been challenging, if we had categorized the vitamin D status using 20 ng/mL as the cut-off. For future studies, use of mentioned equations to generate a contextualized reference range for serum 25(OH) D is recommended.

Among the limitations we faced, we can refer to the relatively sample size, which was mainly imposed due to massive and unexpected devaluation of local currency in Iran that reduced the financial power of the research related to the purchase of laboratory supplies. The research team also decided not to include females into the study design, mainly because almost hundred percent of high school female students in Iran have been receiving monthly high dose of Vitamin D supplements through a national program in all high schools. We also did not assess the sunscreen use or time spent out during sunlight peak in the study population; however, given the relatively narrow time span for data collection and also the narrow age range, we did not expect major differences.

## 5. Conclusions

The findings of this study on male adolescents indicates that serum vitamin D levels of less than 25 ng/mL increases the risk of inflammation and lowers serum HDL level, and, in turn, HDL has a negative correlation with hsCRP level in positive-hsCRP subjects. It can be concluded that low Vitamin D serum levels, through reducing serum HDL levels and induction of inflammation, may contribute in increasing the risk of cardiovascular diseases in this age/sex group but further studies are needed to investigate this causality.

## Figures and Tables

**Table 1:** The anthropometric, dietary, and biochemical variables of the study subjects (*n* = 71).

Variable			Value
Anthropometric variables	Height (cm)		165.67 ± 3.48
	Weight (kg)		63.19 ± 5.16
	BMI (kg/m^2^)		23.00 ± 1.61
	BMI	Normal	61 (85.9)
		Over-weight	10 (14.1)
Dietary variables	Energy (Kcal)		1908.00 ± 171.42
	Carbohydrate (gr)		245.94 ± 41.94
	Fat (gr)		79.68 ± 16.64
	Protein (gr)		58.31 ± 12.62
Biochemical variables	Vitamin D (ng/mL)		23.12 ± 11.31
	TNFR-2 (pg/mL)		18.84 ± 3.74
	IL-10 (pg/mL)		114.82± 26.92
	IL-6 (pg/mL)		124.08 ± 45.36
	hs-CRP (mg/L)		1.28 ± 0.786
Lipid profile	VLDL (mg/dL)		24.36 ± 10.96
	LDL (mg/dL)		86.46 ± 18.32
	TC (mg/dL)		166.97 ± 28.22
	TG (mg/dL)		123.29 ± 54.01
	HDL (mg/dL)		43.88 ± 8.21

BMI: Body Mass Index; hs-CRP: High-Sensitive C-Reactive Protein; IL-6: Interleukin 6; IL-10: Interleukin 10; TNF-α: Tumor Necrosis Factor α; VLDL: Very Low Density Lipoprotein; LDL: Low Density Lipoprotein; TG: Triglyceride; HDL: High Density Lipoprotein; TC: Total Cholesterol. The quantitative and qualitative variables are presented as mean ± SD and frequency (percent), respectively.

**Table 2:** The anthropometric, dietary and biochemical variables in vitamin D deficient and sufficient subjects.

Variable		Vitamin D Status (<25 ng/mL vs. 25 ng/m L≤)		*p*-Value
		Group with Serum Vitamin D below 25 (ng/mL) (SVD < 25) (*n* = 36)	Group with Serum Vitamin D 25 (ng/mL) and above (SVD ≥ 25) (*n* = 35)	
Height (cm)		165.40 ± 3.74	165.92 ± 4.43	0.647 *
Weight (kg)		61.76 ± 4.66	64.51 ± 6.93	0.101 *
BMI (kg/m^2^)		22.57 ± 1.54	22.41 ± 2.12	0.112 *
BMI	Normal	34 (94.4)	27 (77.1)	0.037 **
	Over-weight	2 (5.6)	8 (22.9)	
Vitamin D (ng/mL)		14.50 ± 3.70	31.98 ± 9.47	<0.001 *
TNFR-2 (pg/mL)		20.82 ± 4.13	16.81 ± 1.67	<0.001 *
IL-10 (pg/mL)		111.73 ± 25.42	118.00 ± 28.41	0.330 *
IL-6 (pg/mL)		128.93 ± 56.25	119.09 ± 30.48	0.365 *
hs-CRP (mg/L)		1.46 ± 0.880	1.10 ± 0.636	0.052 *
VLDL (mg/dL)		24.77 ± 11.78	23.94 ± 10.19	0.751 *
LDL (mg/dL)		88.61 ± 19.05	84.25 ± 17.54	0.320 *
TC (mg/dL)		169.33 ± 32.61	164.54 ± 23.08	0.479 *
TG (mg/dL)		126.30 ± 58.27	120.20 ± 49.91	0.637 *
HDL (mg/dL)		37.61 ± 4.01	50.34 ± 6.14	<0.001 *
Dietary energy (Kcal)		1853.98 ± 154.15	1953.90 ± 204.12	0.471
Dietary carbohydrate (gr)		223.01 ± 51.32	254.11 ± 71.45	0.362
Dietary fat (gr)		83.43 ± 17.13	79.12 ± 20.11	0.231
Dietary protein (gr)		53.46 ± 10.07	56.12 ± 13.45	0.193

* *p*-value was reported based on Independent Sample t-test. ** *p*-value was reported based on Chi Square test. The quantitative and qualitative variables are presented as mean ± SD and frequency (percent), respectively. BMI: Body Mass Index; hs-CRP: High Sensitive C-Reactive Protein; IL-6: Interleukin 6; IL-10: Interleukin 10; TNF-α: Tumor Necrosis Factor α; VLDL: Very Low Density Lipoprotein; LDL: Low Density Lipoprotein; TG: Triglyceride; HDL: High Density Lipoprotein; TC: Total Cholesterol; SVD: serum vitamin D.

**Table 3:** The anthropometric, dietary and biochemical variables in hs-CRP positive and negative subjects.

Variable		hs-CRP Status		*p*-Value *
		Positive (*n* = 23)	Negative (*n* = 48)	
Height (cm)		165.18 ± 4.23	166.03 ± 4.00	0.463 *
Weight (kg)		61.68 ± 4.56	64.30 ± 6.81	0.125 *
BMI (kg/m^2^)		22.62 ± 1.73	23.29 ± 1.98	0.211 *
BMI	Normal	29 (96.7)	32 (78)	0.040 **
	Over-weight	1 (3.3)	9 (22)	
Vitamin D (ng/mL)		23.60 ± 12.00	22.11 ± 9.87	0.609 *
TNFR-2 (pg/mL)		18.71 ± 3.86	19.11 ± 3.54	0.676 *
IL-10 (pg/mL)		108.20 ± 22.00	128.62 ± 31.27	0.002 *
IL-6 (pg/mL)		122.42 ± 50.69	127.53 ± 32.24	0.661 *
hs-CRP (mg/L)		1.66 ± 0.653	0.500 ± 0.313	<0.001 *
VLDL (mg/dL)		24.95 ± 10.75	23.13 ± 11.51	0.515 *
LDL (mg/dL)		87.12 ± 18.74	85.08 ± 17.74	0.664 *
TC (mg/dL)		169.75 ± 27.82	161.17 ± 28.77	0.233 *
TG (mg/dL)		127.02 ± 52.62	115.52 ± 57.21	0.405 *
HDL (mg/dL)		44.72 ± 9.15	42.13 ± 5.57	0.215 *
Dietary energy (Kcal)		1976.11 ± 181.06	2010.21 ± 190.41	0.312
Dietary carbohydrate (gr)		276.32 ± 62.11	290.03 ± 82,13	0.231
Dietary fat (gr)		77.34 ± 12.11	79.33 ± 19.34	0.194
Dietary protein (gr)		61.06 ± 11.09	59.34 ± 12.34	0.209

* *p*-value was reported based on Independent Sample t-test. ** *p*-value was reported based on Chi-Square test. The quantitative and qualitative variables are presented as mean± SD and frequency (percent), respectively. BMI: Body Mass Index; hs-CRP: High Sensitive C-Reactive Protein; IL-6: Interleukin 6; IL-10: Interleukin 10; TNF-α: Tumor Necrosis Factor α; VLDL: Very Low Density Lipoprotein; LDL: Low Density Lipoprotein; TG: Triglyceride; HDL: High Density Lipoprotein; TC: Total Cholesterol.

**Table 4:** The correlation of anthropometric, dietary and biochemical variables with serum levels of vitamin D.

Variable		(SVD < 25) (*n* = 36)	(SVD ≥ 25) (*n* = 35)	Total (*n* = 71)
Height (cm)	*p*-value	0.001	0.646	0.854
	r	−0.615	0.093	0.026
Weight (kg)	*p*-value	0.072	0.398	0.101
	r	−0.366	0.169	0.230
BMI (kg/m^2^)	*p*-value	0.992	0.458	0.077
	r	0.002	0.149	0.247
TNFR-2 (pg/mL)	*p*-value	0.875	0.061	0.002
	r	−0.027	0.319	−0.367
IL-10 (pg/mL)	*p*-value	0.091	0.102	0.541
	r	−0.286	−0.281	−0.074
IL-6 (pg/mL)	*p*-value	0.222	0.167	0.108
	r	−0.206	−0.239	−0.193
hs-CRP (mg/L)	*p*-value	0.401	0.823	0.106
	r	−0.144	0.039	−0.193
VLDL (mg/dL)	*p*-value	0.947	0.885	0.731
	r	−0.011	−0.025	−0.041
LDL (mg/dL)	*p*-value	0.716	0.827	0.576
	r	0.063	0.038	−0.067
TC (mg/dL)	*p*-value	0.864	0.249	0.964
	r	−0.029	0.200	−0.005
TG (mg/dL)	*p*-value	0.916	0.934	0.700
	r	0.018	−0.014	−0.047
HDL (mg/dL)	*p*-value	0.165	0.561	<0.001
	r	0.236	0.102	0.657

Statistical analysis was done by Pearson correlation. BMI: Body Mass Index; hs-CRP: High Sensitive C-Reactive Protein; IL-6: Interleukin 6; IL-10: Interleukin 10; TNF-α: Tumor Necrosis Factor α; VLDL: Very Low Density Lipoprotein; LDL: Low Density Lipoprotein; TG: Triglyceride; HDL: High Density Lipoprotein; TC: Total Cholesterol; SVD: serum vitamin D.

**Table 5:** The correlation of anthropometric, dietary, and biochemical variables with serum levels of hs-CRP.

Variable		hs-CRP Negative (*n* = 23)	hs-CRP Positive (*n* = 48)	Total (*n* = 71)
Height (cm)	*p*-value	0.150	0.823	0.588
	r	0.269	−0.051	−0.077
Weight (kg)	*p*-value	0.375	0.568	0.197
	r	0.168	−0.129	−0.182
BMI (kg/m^2^)	*p*-value	0.783	0.697	0.267
	r	0.052	−0.088	−0.157
TNFR-2 (pg/mL)	*p*-value	0.206	0.138	0.310
	r	0.274	0.217	0.122
IL-10 (pg/mL)	*p*-value	0.315	0.981	0.070
	r	0.219	0.003	−0.216
IL-6 (pg/mL)	*p*-value	0.485	0.137	0.351
	r	0.153	0.218	0.112
Vitamin D (ng/mL)	*p*-value	0.020	0.031	0.106
	r	−0.481	−0.311	−0.193
VLDL (mg/dL)	*p*-value	0.952	0.749	0.800
	r	0.013	-0.047	0.031
LDL (mg/dL)	*p*-value	0.194	0.884	0.492
	r	0.281	0.022	0.083
TC (mg/dL)	*p*-value	0.219	0.872	0.315
	r	0.267	−0.024	0.121
TG (mg/dL)	*p*-value	0.959	0.605	0.804
	r	0.011	−0.076	0.030
HDL (mg/dL)	*p*-value	0.128	0.020	0.273
	r	−0.327	−0.335	−0.132

Statistical analysis was done by Pearson correlation. BMI: Body Mass Index; hs-CRP: High Sensitive C-Reactive Protein; IL-6: Interleukin 6; IL-10: Interleukin 10; TNF-α: Tumor Necrosis Factor α; VLDL: Very Low Density Lipoprotein; LDL: Low Density Lipoprotein; TG: Triglyceride; HDL: High Density Lipoprotein; TC: Total Cholesterol.

## References

[1] Hill T.R., Aspray T.J. (2017). The role of vitamin D in maintaining bone health in older people. Ther. Adv. Musculoskelet. Dis..

[2] Wang H., Chen W., Li N., Yin X., Zhang X., Olsen N., Zheng S.G. (2017). Vitamin D and Chronic Diseases. Aging Dis..

[3] Rolf L., Muris A.-H., Hupperts R., Damoiseaux J. (2016). Illuminating vitamin D effects on B-cells—The multiple sclerosis perspective. Immunology.

[4] Hoe E., Nathanielsz J., Toh Z.Q., Spry L., Marimla R., Balloch A., Mulholland E.K., Licciardi P. (2016). Anti-Inflammatory Effects of Vitamin D on Human Immune Cells in the Context of Bacterial Infection. Nutrients.

[5] Jacobs E.T., Kohler L.N., Kunihiro A.G., Jurutka P. (2016). Vitamin D and Colorectal, Breast, and Prostate Cancers: A Review of the Epidemiological Evidence. J. Cancer.

[6] Christodoulou S., Goula T., Ververidis A., Drosos G. (2012). Vitamin D and Bone Disease. BioMed Res. Int..

[7] Kota S., Jammula S., Kota S., Meher L., Modi K. (2013). Correlation of vitamin D, bone mineral density and parathyroid hormone levels in adults with low bone density. Indian J. Orthop..

[8] Lips P., Goldsmith D., De Jongh R. (2017). Vitamin D and osteoporosis in chronic kidney disease. J. Nephrol..

[9] Welsh P., Sattar N. (2014). Vitamin D and chronic disease prevention. BMJ.

[10] Ashtari F., Toghianifar N., Esfahani S.H.Z., Mansourian M. (2015). Short-Term Effect of High-Dose Vitamin D on the Level of Interleukin 10 in Patients with Multiple Sclerosis: A Randomized, Double-Blind, Placebo-Controlled Clinical Trial. Neuroimmunomodulation.

[11] Heine G., Niesner U., Chang H.-D., Steinmeyer A., Zügel U., Zuberbier T., Radbruch A., Worm M. (2008). 1,25-dihydroxyvitamin D3promotes IL-10 production in human B cells. Eur. J. Immunol..

[12] Slavin A.J., Maron R., Weiner H.L. (2001). Mucosal administration of IL-10 enhances oral tolerance in autoimmune encephalomyelitis and diabetes. Int. Immunol..

[13] Mangin M., Sinha R., Fincher K. (2014). Inflammation and vitamin D: The infection connection. Inflamm. Res..

[14] Liu W., Zhang L., Xu H.-J., Li Y., Hu C.-M., Yang J.-Y., Sun M. (2018). The Anti-Inflammatory Effects of Vitamin D in Tumorigenesis. Int. J. Mol. Sci..

[15] Galler J.R., Koethe J.R., Yolken R.H. (2017). Neurodevelopment: The Impact of Nutrition and Inflammation during Adolescence in Low-Resource Settings. Pediatrics.

[16] Vohr B.R., Davis E.P., Wanke C.A., Krebs N.F. (2017). Neurodevelopment: The Impact of Nutrition and Inflammation during Preconception and Pregnancy in Low-Resource Settings. Pediatrics.

[17] Wajant H., Siegmund D. (2019). TNFR1 and TNFR2 in the Control of the Life and Death Balance of Macrophages. Front. Cell Dev. Biol..

[18] Dobnig H., Pilz S., Scharnagl H., Renner W., Seelhorst U., Wellnitz B., Kinkeldei J., Boehm B.O., Weihrauch G., März W. (2008). Independent Association of Low Serum 25-Hydroxyvitamin D and 1,25-Dihydroxyvitamin D Levels With All-Cause and Cardiovascular Mortality. Arch. Intern. Med..

[19] Li Y.C., Kong J., Wei M., Chen Z.F., Liu S.Q., Cao L.P. (2002). 1,25-Dihydroxyvitamin D(3) is a negative endocrine regulator of the renin-angiotensin system. J. Clin. Investig..

[20] Lee J.H., O’Keefe J.H., Bell D., Hensrud D.D., Holick M.F. (2008). Vitamin D deficiency an important, common, and easily treatable cardiovascular risk factor?. J. Am. Coll. Cardiol..

[21] Wang T.J., Pencina M.J., Booth S.L., Jacques P.F., Ingelsson E., Lanier K., Benjamin E.J., D’Agostino R.B., Wolf M., Vasan R.S. (2008). Vitamin D deficiency and risk of cardiovascular disease. Circulation.

[22] Jorde R., Figenschau Y., Hutchinson M., Emaus N., Grimnes G. (2010). High serum 25-hydroxyvitamin D concentrations are associated with a favorable serum lipid profile. Eur. J. Clin. Nutr..

[23] Wang Y., Si S., Liu J., Wang Z., Jia H., Feng K., Sun L., Song S. (2016). The Associations of Serum Lipids with Vitamin D Status. PLOS ONE.

[24] Kelishadi R., Farajzadegan Z., Bahreynian M. (2014). Association between vitamin D status and lipid profile in children and adolescents: A systematic review and meta-analysis. Int. J. Food Sci. Nutr..

[25] Mirmiran P., Hosseini-Esfahani F., Mehrabi Y., Hedayati M., Azizi F. (2009). Reliability and relative validity of an FFQ for nutrients in the Tehran Lipid and Glucose Study. Public Health Nutr..

[26] Kennel K.A., Drake M.T., Hurley D.L. (2010). Vitamin D Deficiency in Adults: When to Test and How to Treat. Mayo Clin. Proc..

[27] Pais P., Kamath D.Y., Xavier D., Sigamani A. (2015). High sensitivity C-reactive protein (hsCRP) & cardiovascular disease: An Indian perspective. Indian J. Med. Res..

[28] Plichta S.B., Kelvin E.A., Munro B.H. (2012). Munro’s Statistical Methods for Health Care Research.

[29] Spotila L.D., Rodriguez H., Koch M., Adams K., Caminis J., Tenenhouse H.S., Tenenhouse A. (2000). Association of a Polymorphism in the TNFR2 Gene with Low Bone Mineral Density. J. Bone Miner. Res..

[30] Barker T., Brown K.B., E Rogers V. (2017). Soluble TNF Receptors are Modulated by Vitamin D Status but not by Acute Perturbations in 25-Hydroxyvitamin D Following A Bolus of Supplemental Vitamin D. J. Cytokine Biol..

[31] Bertolini D.R., Nedwin G.E., Bringman T.S., Smith D.D., Mundy G.R. (1986). Stimulation of bone resorption and inhibition of bone formation in vitro by human tumour necrosis factors. Nature.

[32] Nguyen L., E Dewhirst F., Hauschka P.V., Stashenko P. (1991). Interleukin-1 beta stimulates bone resorption and inhibits bone formation in vivo. Lymphokine Cytokine Res..

[33] Stashenko P., Dewhirst F.E., Rooney M.L., Desjardins L.A., Heeley J.D. (2009). Interleukin-1β is a potent inhibitor of bone formation in vitro. J. Bone Miner. Res..

[34] Abu-Amer Y. (2000). Tumor necrosis factor receptors types 1 and 2 differentially regulate osteoclastogenesis. J. Biol. Chem..

[35] Opal S.M., DePalo V.A. (2000). Anti-Inflammatory Cytokines. Chest.

[36] Takemura M., Matsumoto H., Niimi A., Ueda T., Matsuoka H., Yamaguchi M., Jinnai M., Muro S., Hirai T., Ito Y. (2006). High sensitivity C-reactive protein in asthma. Eur. Respir. J..

[37] Seyrek N., Karayaylali I., Balal M., Paydas S., Aikimbaev K., Çetíner S., Seydaoglu G. (2005). Is there any relationship between serum levels of interleukin-10 and atherosclerosis in hemodialysis patients?. Scand. J. Urol. Nephrol..

[38] Ngo D.T., Sverdlov A., McNeil J.J., Horowitz J.D. (2010). Does Vitamin D Modulate Asymmetric Dimethylarginine and C-Reactive Protein Concentrations?. Am. J. Med..

[39] Chen N., Wan Z., Han S., Li B., Zhang Z., Qin L.-Q. (2014). Effect of Vitamin D Supplementation on the Level of Circulating High-Sensitivity C-Reactive Protein: A Meta-Analysis of Randomized Controlled Trials. Nutrients.

[40] Rahimi-Ardabili H., Gargari B.P., Farzadi L. (2012). Effects of vitamin D on cardiovascular disease risk factors in polycystic ovary syndrome women with vitamin D deficiency. J. Endocrinol. Investig..

[41] Nam G.E., Kim D.-H., Cho K.H., Park Y.G., Han K.D., Kim S.M., Lee S.H., Ko B.J., Kim M.J. (2012). 25-Hydroxyvitamin D insufficiency is associated with cardiometabolic risk in Korean adolescents: the 2008–2009 Korea National Health and Nutrition Examination Survey (KNHANES). Public Health Nutr..

[42] Cho H.-J., Kang H.-C., Choi S.-A., Ju Y.-C., Lee H.-S., Park H.-J. (2005). The possible role of Ca2+ on the activation of microsomal triglyceride transfer protein in rat hepatocytes. Biol. Pharm. Bull..

[43] Christensen R., Lorenzen J.K., Svith C.R., Bartels E.M., Melanson E.L., Saris W.H., Tremblay A., Astrup A. (2009). Effect of calcium from dairy and dietary supplements on faecal fat excretion: A meta-analysis of randomized controlled trials. Obes. Rev..

[44] Vaskonen T., Mervaala E., Sumuvuori V., Seppanen-Laakso T., Karppanen H. (2002). Effects of calcium and plant sterols on serum lipids in obese Zucker rats on a low-fat diet. Br. J. Nutr..

[45] Song S.J., Si S., Liu J., Chen X., Zhou L., Jia G., Liu G., Niu Y., Wu J., Zhang W. (2012). Vitamin D status in Chinese pregnant women and their newborns in Beijing and their relationships to birth size. Public Health Nutr..

[46] Zittermann A., Frisch S., Berthold H., Götting C., Kuhn J., Kleesiek K., Stehle P., Koertke H., Koerfer R. (2009). Vitamin D supplementation enhances the beneficial effects of weight loss on cardiovascular disease risk markers. Am. J. Clin. Nutr..

[47] Karnchanasorn R., Ou H.Y., Chiu K.C. (2012). Plasma 25-hydroxyvitamin D levels are favorably associated with beta-cell function. Pancreas.

[48] Howard B.V. (1999). Insulin resistance and lipid metabolism. Am. J. Cardiol..

[49] Tai E.S., Emmanuel S.C., Chew S.K., Tan B.Y., Tan C. (1999). Isolated low HDL cholesterol: An insulin-resistant state only in the presence of fasting hypertriglyceridemia. Diabetes.

[50] Jiang W., Miyamoto T., Kakizawa T., Nishio S., Oiwa A., Takeda T., Suzuki S., Hashizume K., Miyamoto T., Kakizawa T. (2006). Inhibition of LXRalpha signaling by vitamin D receptor: Possible role of VDR in bile acid synthesis. Biochem. Biophys. Res. Commun..

[51] Fraunberger P., Schaefer S., Werdan K., Walli A.K., Seidel D. (1999). Reduction of Circulating Cholesterol and Apolipoprotein Levels during Sepsis. Clin. Chem. Lab. Med..

[52] Murch O., Collin M., Hinds C.J., Thiemermann C. (2006). Lipoproteins in inflammation and sepsis. I. Basic science. Intensiv. Care Med..

[53] Shor R., Wainstein J., Oz D., Boaz M., Matas Z., Fux A., Halabe A. (2007). Low HDL levels and the risk of death, sepsis and malignancy. Clin. Res. Cardiol..

[54] Thompson P.A., Berbée J.F., Rensen P.C., Kitchens R.L. (2008). Apolipoprotein A-II augments monocyte responses to LPS by suppressing the inhibitory activity of LPS-binding protein. Innate Immun..

[55] Chang S.-W., Lee H.-C. (2019). Vitamin D and health - The missing vitamin in humans. Pediatr. Neonatol..

[56] Ferrari D., Lombardi G., Banfi G. (2017). Concerning the vitamin D reference range: Pre-analytical and analytical variability of vitamin D measurement. Biochem. Med..

[57] Napoli N., Strollo R., Sprini D., Maddaloni E., Rini G.B., Carmina E. (2014). Serum 25-OH Vitamin D in relation to Bone Mineral Density and Bone Turnover. Int. J. Endocrinol..

